# Radiotherapy in combination with systemic therapies for brain metastases: current status and progress

**DOI:** 10.20892/j.issn.2095-3941.2020.0109

**Published:** 2020-12-15

**Authors:** Lei Liu, Wanqi Chen, Ruopeng Zhang, Yuekun Wang, Penghao Liu, Xin Lian, Fuquan Zhang, Yu Wang, Wenbin Ma

**Affiliations:** 1 Department of Neurosurgery, Peking Union Medical College Hospital, Chinese Academy of Medical Sciences and Peking Union Medical College, Beijing 100730, China; 2 Department of Radiotherapy, Peking Union Medical College Hospital, Chinese Academy of Medical Sciences and Peking Union Medical College, Beijing 100730, China

**Keywords:** Brain metastases, radiotherapy, targeted therapy, immunotherapy, systemic therapy

## Abstract

Brain metastases (BMs) are the most common cause of intracranial neoplasms in adults with poor prognosis. Most BMs originate from lung cancer, breast cancer, or melanoma. Radiotherapy (RT), including whole brain radiotherapy (WBRT) and stereotactic radiation surgery (SRS), has been widely explored and is considered a mainstay anticancer treatment for BMs. Over the past decade, the advent of novel systemic therapies has revolutionized the treatment of BMs. In this context, there is a strong rationale for using a combination of treatments based on RT, with the aim of achieving both local disease control and extracranial disease control. This review focuses on describing the latest progress in RT as well as the synergistic effects of the optimal combinations of RT and systemic treatment modalities for BMs, to provide perspectives on current treatments.

## Introduction

Brain metastases (BMs), the most common intracranial neoplasms in adults with invasive cancer, cause a decline in neurological function and overall quality of life (QoL), and may result in mortality from recurrent or untreatable lesions. Approximately 20%–45% of patients with tumors are diagnosed with BMs in their lifetime^1^. Most BMs originate from lung cancer, breast cancer, or melanoma^2^. The current standard management for BMs, consisting of a multimodal approach including surgery and/or radiotherapy (RT), systemic therapy, and symptomatic therapy, remains controversial and ineffective^3^.

RT, in the form of whole brain radiotherapy (WBRT) and stereotactic radiation surgery (SRS), is considered a mainstay anticancer modality in the treatment of BMs from solid tumors. RT provides better local tumor control with relatively fewer systemic adverse effects than chemotherapy^4,5^. Over the past decade, the advent of novel systemic therapies, including chemotherapy, targeted therapy (TT), and immunotherapy (IT), has revolutionized systemic therapy for several malignancies. Consequently, the combination of RT and systemic therapies has been investigated with the aim of achieving both local and extracranial disease control, and possibly improvement in overall survival (OS).

Given the lack of updated guidelines for the treatment of patients with BMs, particularly reflecting the introduction of new therapies, this review aims to describe the effects of combining RT with other systemic treatment modalities for BMs, and to provide perspectives on current treatments.

## RT for brain metastases

WBRT is primarily used as an alternative therapy and a palliative therapy for BM when local treatment cannot be applied^6^. Intracranial progression can be completely or partially controlled after WBRT with a low risk of local recurrence and new metastases^7–10^. However, whether WBRT should be routinely used in patients with BMs remains controversial, because the OS or QoL have not been found to improve after WBRT, whereas impaired cognitive function and decreased QoL have been observed^9,11–14^.

Compared with WBRT, SRS results in a similar OS with improvement in cognitive function; previous studies have indicated that SRS can be used as a standard therapy for patients with limited BM after surgery^15–18^.

For extensive BM, usually defined by the presence of 5 to 10 metastatic lesions, WBRT is the standard treatment recommended in most guidelines^6^. However, a group of Japanese observational studies have indicated that the performance of SRS alone for extensive BMs is not inferior to that of SRS for 2 to 4 metastatic lesions, and does not result in significant differences in OS^4,19,20^. Because SRS has relatively fewer adverse effects, these studies have recommended SRS as a treatment option for extensive BM. Whether WBRT plus SRS should be used in patients with BMs is currently unclear. The results of 3 phase III randomized controlled trials (RCTs) have shown that WBRT after SRS, compared with SRS alone, decreases the intracranial recurrence rate, but causes more significant cognitive impairment and does not result in a difference in OS^9,12,14^.

Therefore, SRS alone should be strongly recommended in patients with limited BMs (1–4) or those younger than 50 years who are about to receive active systemic therapy^13,14^. Patients with a high risk of central failure might benefit from WBRT combined with SRS.

Early studies have recommended postoperative RT^7,9^, but extremely few studies have defined the optimal therapeutic time interval (TTI) between surgery and RT, and TTIs vary among trials^21^. Recent retrospective studies have reported that preoperative SRS significantly decreases the incidence of meningeal metastases and symptomatic radiation necrosis compared with postoperative SRS, and the OS of preoperative SRS is even not inferior^22,23^. Retrospective studies have reported that hyperfractionation results in better local control compared with that of lower dose regimens and is associated with a lower risk of necrosis^24^.

## RT combined with chemotherapy

Systemic therapy, an emerging modality, has also shown a great therapeutic potential and has demonstrated activity in the brain. Although most chemotherapeutic drugs have poor penetration of the blood-brain barrier (BBB), RT might help disrupt the BBB to allow the drugs to penetrate into the BMs^25^. Owing to their poor BBB penetration, chemotherapeutic drugs are generally not preferred as the first-line treatment but instead are used in addition to local treatment^7^. Recently, several studies have evaluated temozolomide (TMZ), which can penetrate the BBB, and have suggested that TMZ has therapeutic effects in recurrent and progressive BMs. Phase II trials of RT combined with TMZ have suggested that this combination, compared with TMZ alone, significantly improves local control but not the OS^25–27^.

For non-small cell lung cancer (NSCLC) BMs, platinum, either alone or in combination with other drugs, has been widely used as a standard treatment regimen before or after RT. Compared with RT alone, the combination of RT and chemotherapy improves the local response rate of BMs but not the OS^28^. However, there is no standard chemotherapy regimen for patients with BMs from breast cancer or melanoma, for which RT, chemotherapy, or chemoradiotherapy has shown only a modest effect on survival^6^.

## RT combined with TT

TT is a topic of interest in BM treatment research. The response rates of specific molecular subtypes to targeted drugs are higher than those to cytotoxic chemotherapy drugs. However, owing to their poor prognosis, patients with BMs have long been excluded from clinical trials evaluating systemic therapies in primary solid tumors. Thus, the role of systemic therapies, particularly in combination with RT for BMs, is poorly understood. The use of a combination of RT and TT might be effective, but the safety and exact efficacy in patients with various types of BMs remain unclear. Most reported studies have been retrospective or phase I/II clinical trials with small samples and inconsistent conclusions. Hence, high-level evidence, such as from large-scale phase III RCTs, is urgently needed. Additionally, previous studies have lacked stratification of BMs according to factors such as mutational status. The dose fraction of RT, the timing of administration, and the dose of combined targeted drugs must also be further explored.

### Lung cancer

Targeted drugs for lung cancer, mostly NSCLC, mainly include EGFR-tyrosine kinase inhibitors (EGFR-TKIs) and ALK-tyrosine kinase inhibitors (ALK-TKIs). These drugs have shown a high response rate and safety for EGFR- or ALK-mutant NSCLC BMs^29–33^. A phase III clinical trial (PL03.05: BRAIN) has reported that icotinib is well tolerated and results in a significantly longer OS than chemoradiotherapy^34^.

Many pre-clinical and *in vitro* studies have reported the role of EGFR in regulating radiosensitivity. EGFR inhibitors (EGFRi) can decrease the radioresistance of tumor cells, and EGFR-mutant cell lines show higher radiosensitivity than wild-type cells^35–37^. Moreover, RT may in turn disrupt the BBB and consequently increase the ability of EGFR-TKIs to permeate the BBB^38^. Hence, the use of a combination of RT and TKIs might be very effective because of their synergistic effects. However, the results of current clinical studies on combination therapy are contradictory; hence, no firm conclusions have been reached^39–43^. Several phase II trials have suggested that WBRT combined with TKIs is well tolerated and either does not increase or even decreases neurocognitive toxicity^44–47^. Another study has suggested that WBRT combined with TKIs increases the incidence of toxic responses or results in no improvement in OS. The RTOG 0320 (phase III) trial has concluded that grade 3 to 5 toxicity significantly increases and that the survival is not improved after the addition of erlotinib to WBRT and SRS^48^. A multicenter retrospective study comparing SRS followed by EGFR-TKI, WBRT followed by EGFR-TKI, and EGFR-TKI followed by SRS or WBRT has suggested that patients with NSCLC BMs with EGFR mutations who receive SRS followed by EGFR-TKI have the longest OS and are able to avoid the potential neurocognitive sequelae of WBRT^49^.

Preclinical studies have also reported that concurrent RT plus ALK-TKIs synergistically affect tumor growth and microvessel density^50,51^, thus potentially resulting in better local control. A multi-institutional retrospective analysis has shown prolonged OS in patients with ALK-rearranged NSCLC BMs treated with SRS plus ALK inhibitors^52^. However, neither prospective nor more convincing retrospective clinical data have been reported.

Notably, the previous studies have enrolled mostly patients with both wild-type and mutant NSCLC and have lacked stratification based on mutation status, thus potentially explaining the inconsistent results across studies. A single-arm phase II trial has reported that most patients with EGFR mutations show an impressive intracranial response rate when erlotinib is added to WBRT^45^. Another phase II trial has reported that most patients carry wild-type EGFR and has concluded that there is no improvement in the neurological PFS or OS in patients with multiple BMs^53^. Patients with EGFR or ALK mutations show significantly better efficacy than those with wild-type EGFR or ALK^28,47^. Therefore, future studies should be conducted in subgroups according to the mutation status.

### Breast cancer

The 2018 ASCO guidelines recommend RT as a standard treatment for breast cancer BMs (BCBMs)^54^. Lapatinib plus capecitabine can be used as the initial treatment for BMs in breast cancer before RT^55^. Compared with chemotherapy, HER2-targeted drugs and targeted plus chemical drugs result in higher response rates and improved survival^56^. Pre-clinical studies have shown that the HER2-targeted antibody-drug conjugate T-DM1 may increase radiosensitivity, whereas RT interferes with the permeability of the BBB and enhances breast HER2/neu expression, thus potentially sensitizing cells to the antiproliferative effects of anti-HER2 therapy^57^. To date, few clinical trials have been conducted to assess the efficacy of RT plus TT in BCBMs, all of which are phase I/II trials with low response rates and few positive results.

Two retrospective studies on HER2^+^ BCBMs conducted by the Curie Institute support the safety and effectiveness of concurrent WBRT and trastuzumab as a radiosensitizer, but further research is needed^58^. Yomo et al.^59^ have retrospectively analyzed 40 HER2^+^ breast cancer patients receiving SRS with or without lapatinib. The 1-year local tumor control rate in the lapatinib group was significantly higher than that that in the non-lapatinib group, thus suggesting that lapatinib has a synergistic effect with SRS.

### Melanoma

Vemurafenib, a BRAF inhibitor (BRAFi), is effective in BMs from BRAF-mutated melanoma^60–62^. Pre-clinical studies have suggested that the MAPK pathway is often upregulated in cancer cells and is activated by exposure to ionizing radiation; BRAFi treatment can increase the radiosensitivity of tumor cells by blocking this pathway. In patients with a BRAF mutation, the radiotherapeutic response can be increased when treated with ionizing radiation combined with TT^63,64^. The mechanism underlying this improvement is unclear and remains under investigation, and high-level evidence is required.

A prospective study involving 80 patients with melanoma brain metastases (MBMs) has indicated an improvement in OS in BRAF-mutant patients treated with SRS combined with BRAFi^65^. Other studies have found that the administration of BRAFi±MEK inhibitor (BRAFi±MEKi) after RT is safe and effective in patients with MBMs, thus indicating that the combination of the 2 treatments may have a synergistic and superior clinical effect^62,66–69^. Although the timing and sequence of the 2 therapies suggest a significant synergistic effect, the optimal timing and sequence remain unknown, particularly in patients with multiple BMs.

### Other targeted therapies

Other targeted sites are also under investigation, such as those targeted by anti-angiogenic agents. Antiangiogenic agents, including bevacizumab, inhibit tumor angiogenesis by blocking VEGF and promote tumor ischemic necrosis, thus making them a potential RT sensitizer. RT in turn affects the expression of angiogenic factors and tumor growth factors such as VEGF, Ang-2, and Ang-1, and their receptor Tie-2. In mouse models, low-dose radiation promotes tumor growth and metastasis by activating VEGFR2^70^. A single-arm phase I REBECA trial including 19 patients with unresectable solid tumor BMs and has indicated that bevacizumab plus WBRT treatment is tolerable but requires further evaluation^71^. Higher-level evidence is currently lacking.

## RT combined with IT

IT, particularly monoclonal antibodies targeting immune checkpoint pathways (PD-1/PD-L1 and CTLA-4), improves the OS of patients with advanced tumors, such as melanoma and NSCLC, which frequently develop BMs^72^. Moreover, a synergistic effect has been observed when RT is administered with IT in patients with BM. Hence, more efforts are focusing on evaluating the effects of RT combined with ITs, including antibody-based immune checkpoint blockers, cancer vaccines, and T-cell therapies.

RT can induce both antitumor and pro-tumor immune responses in the tumor microenvironment (TME), and the balance of these responses must be further investigated. For the antitumor immune response induced by RT, growing evidence suggests that RT locally interacts with the immune system by inducing the production of immune factors and tumor antigens in the irradiated TME (ITME), which can individually or synergistically prime the immune system, as well as by activating immune cells^73–76^. Recent studies have also demonstrated that the recruitment of dendritic cells and several other immune effector cell types is mediated by cytokines, such as CXCL16, TNFα, IL-1β, and IL-6, that are secreted by tumor cells after radiation^77^. Moreover, RT administered with IT has a synergistic effect not only at the irradiated target but also at distant sites, owing to the immune regulation initiated by the local TME (abscopal effect)^78^.

Regarding pro-tumor immune regulation, RT can also upregulate radiation resistance in a manner mediated by radiation-associated antigenic proteins, such as PD-1/PD-L1 and CD47, through the activation of NF-κB^79–81^. RT increases IFNγ produced by CD8^+^ T cells, thus resulting in enhanced PD-L1 expression on tumor cells^82,83^. CTLA-4, which is predominantly expressed by Treg cells, is an immune checkpoint inhibitor (ICI) together with PD-1/PD-L1. Its high expression in cancer cells inhibits immune effector cell activation by increasing CTLA4-CD80/86 binding^84^. Finally, targeted IT used to block these immunosuppressive pathways can enhance the response rate when it is combined with RT.

### Lung cancer

Several retrospective studies have suggested that the use of a combination of IT and RT for BMs from NSCLC might be a safe strategy with promising activity. However, the safety and efficacy of IT administered with brain RT have not been fully clarified.

CIs such as PD-1/PD-L1 inhibitors are the primary treatment for BMs from lung cancer. Hubbeling et al.^85^ have determined the safety of RT combined with ICIs in patients with advanced NSCLC BMs. They have found that treatment with ICI and cranial RT does not significantly increase RT-related adverse events, thus suggesting that the use of RT and ICIs is well tolerated. Two other retrospective studies including 66 patients with NSCLC have characterized the effects of concurrent RT and ICIs on survival outcomes and safety and have not observed increased adverse events in patients with BMs from different solid tumors^86,87^. Schapira et al.^88^ have retrospectively reviewed the outcomes of 37 patients with BMs from NSCLC treated with PD-1 pathway inhibitors (83.8% nivolumab, 10.8% atezolizumab, and 5.4% pembrolizumab) and SRS. The patients treated with concurrent SRS and PD-1 pathway inhibitors had longer OS than those treated with SRS before or after IT.

NSCLC comprises a higher percentage of BMs than SCLC, and recent studies have suggested that SCLC has a higher biological propensity for the central nervous system^89^. However, for SCLC, unlike NSCLC, there are very few indications of SCLC-specific targets^90^. Trials using antibodies targeting PD-1 (nivolumab) and CTLA4 (ipilimumab) on BMs from SCLC are underway.

### Breast cancer

Hu et al.^91^ have found that the overall response rate of breast cancers to IT is only 19%. Dewan et al.^92^ have found that fractionated RT combined with anti-CTLA4 treatment not only delays the growth of primary tumors but also induces an abscopal effect with enhanced CD8^+^ T cells in mouse breast models. However, metastasis to the brain from breast cancer remains a major clinical challenge. Recently, chimeric antigen receptor (CAR)-based immune therapy for the treatment of BCBMs has garnered attention. Priceman et al.^93^ have optimized HER2-CAR T cells containing either CD28 or 4-1BB intracellular costimulatory signaling domains and have demonstrated robust antitumor efficacy after the regional intraventricular delivery of HER2-CAR T cells for the treatment of HER2^+^ breast cancer metastasis to the brain in a mouse model. Further studies are warranted to validate this approach.

### Melanoma

In the past decade, several new systemic drugs have been introduced, including IT with checkpoint inhibitors such as anti-CTLA4 antibodies [ipilimumab (IPI)]^94,95^, anti-PD1 antibodies (nivolumab and pembrolizumab)^96,97^, or a combination of these drugs^98^, and promising results have been observed in patients with melanoma. However, patients with MBMs have been excluded from clinical trials evaluating the efficacy of IT in melanoma. Hence, the role of IT in MBMs is poorly understood, particularly in combination with RT.

Opijnen et al.^99^ have emphasized the role of a combination of RT and IT for MBMs and have found that the combination of IT and SRS is highly effective, with a weighted median OS of 17.4 months. A phase I study aiming to determine the maximum tolerable dose and safety of ipilimumab with SRS or WBRT in patients with MBMs has found that SRS and IT are more effective^100^. The study has also demonstrated the safety of concurrent ipilimumab 10 mg/kg with SRS.

However, other studies evaluating the efficacy of a combination of RT and IT in MBMs have been retrospective, and no prospective randomized studies have been performed. Among these retrospective studies, Stokes et al.^101^ have examined the largest sample size from the National Cancer Database, with 185 patients receiving both RT and IT. The study suggests that adding IT to RT for MBMs is associated with prolonged survival, with a median of 10.8 months. The factors associated with this improved survival have been found to include stereotactic RT, chemotherapy, and IT. Fang et al.^102^ have evaluated 137 patients with MBM treated with SRS and anti-CTLA-4 and/or anti-PD-1 antibodies, and have reported a median OS of 16.9 months. They have also characterized radiation necrosis after IT and SRS and demonstrated that the IT type and timing proximity to SRS are not associated with radiation necrosis risk. In addition to the survival benefit, the relative benefit in local control after a combination of RT and IT has been found to be significant^103,104^.

A previous systematic review has reported the outcomes of patients treated with IT and RT for BMs, including 33 studies, 28 of which were associated with MBMs. The pooled median OS from the start of treatment was 15.9 months, and the 1-year OS rate was 55.2%. Moreover, RT administered before or concurrently with IT may provide better results than inverse sequencing^105^.

However, more RCTs or prospective studies are warranted to generate proper evidence that can be used to change the standard of care for patients with MBMs.

All the clinical trials for the combined therapies mentioned above are summarized in **Table 1**.

**Table 1:** Characteristics of clinical trials examining treatments for brain metastases (RT+chemo, RT+TT, and RT+IT)

Study	Study design	Number of patients	Primary tumor	Treatment	RT type	Drugs	Median OS (m)	Other results
**RT+chemo**								
Antonadou et al., 2002^25^	Phase II trial	52	Solid tumor	RT+chemo *vs.* RT alone	WBRT	TMZ		WBRT+TMZ: ORR significantly improved (*P* = 0.017)
Verger et al., 2005^26^	Phase II trial	82	Solid tumor	RT+chemo *vs.* RT alone	WBRT	TMZ		Percentage of patients with PFS at 90 days: WBRT (54%) *vs.* WBRT+TMZ-72%
Chua et al., 2010^27^	Phase II trial	70	NSCLC	RT+chemo *vs.* RT alone	WBRT	TMZ	WBRT+TMZ *vs.* WBRT: 4.4, 5.7	Median time to CNS progression: WBRT+TMZ (3.1 m) *vs.* WBRT (3.8 m)
**RT+TT**								
Chen et al., 2016^40^	Retrospective study	132	EGFR-mutated lung adenocarcinoma	RT+TT *vs.* TT alone	WBRT	Gefitinib or erlotinib	WBRT+TT* vs.* TT: 48.0, 41.1	Intracranial ORR: significantly higher in the WBRT+TT group (67.9%) than the TT group (39.2%) (*P* = 0.001)
Jiang et al., 2016^41^	Retrospective study	230	EGFR-mutant NSCLC	RT+TT *vs.* TT alone	WBRT	EGFR-TKI	WBRT+TT *vs.* TT: 21.6, 26.4	iPFS and systemic PFS: WBRT+TT (6.9 m, 7.5 m) *vs.* TT (7.4 m, 7.9 m)
Magnuson et al., 2017^49^	Retrospective study	351	EGFR-mutant NSCLC	SRS followed by TT, WBRT followed by TT, or TT followed by SRS/WBRT	WBRT/SRS	EGFR-TKI	SRS, WBRT, and EGFR-TKI cohorts: 46, 30, and 25	
Lee et al., 2014^53^	RCT	80	NSCLC	RT+TT *vs.* RT+placebo	WBRT	Erlotinib	TT *vs.* placebo: 3.4, 2.9	Median iPFS: 1.6 m in both arms
Pesce et al., 2012^44^	Phase II trial	59	NSCLC	RT+TT *vs.* RT+chemo	WBRT	Gefitinib *vs.* TMZ	Gefitinib *vs.* TMZ: 6.3, 4.9	
Welsh et al., 2013^45^	Single-arm phase II trial	40	NSCLC	RT+TT	WBRT	Erlotinib	11.8	ORR: 86%
Fan et al., 2015^47^	Single-arm phase II trial	20	NSCLC	RT+TT	WBRT	Icotinib	14.6	ORR: 80.0%
Sperduto et al., 2013^48^	Phase III trial	126	NSCLC	RT+TT *vs.* RT+chemo *vs.* RT alone	WBRT+SRS	Erlotinib *vs.* TMZ	WBRT+SRS, WBRT+SRS+TMZ, and WBRT+SRS+ETN: (13.4, 6.3, and 6.1)	Rates of serious (grade 3–5) toxicity: 11%, 41%, and 49%
Johung et al., 2016^52^	Retrospective study	90	ALK-rearranged NSCLC	RT+TT	WBRT or SRS	ALK-TKI	49.5	Median iPFS: 11.9 m
Chargari et al., 2011^58^	Retrospective study	31	EGFR-2-positive breast cancer	RT+TT	WBRT	Trastuzumab	18	Median iPFS: 10.5 m; clinical response: 87.1%
Yomo et al., 2013^59^	Retrospective study	40	HER2-overexpressing breast cancer	RT+TT *vs.* RT alone	SRS	Lapatinib		Rate of 1-year LC: RT+TT (86%) *vs.* RT (69)
Wolf et al., 2016^65^	Prospective study	80	Melanoma	RT+TT *vs.* RT alone	SRS	BRAF inhibitor	SRS+TT *vs.* SRS: 11.2, 6.7	Median iPFS: SRS+TT(3.9 m) *vs.* SRS (1.7 m)
**RT+IT**								
Hubbeling et al., 2018^85^	Retrospective study	50	NSCLC	RT+IT	WBRT, SRS, PBI	NIVO, PEMBRO or ATEZO		Grade ≥3 AEs in 8% of ICI-naive patients *vs.* in 9% of ICI-treated patients for SRS (*P* = 1.00)
Chen et al., 2018^87^	Retrospective study	37	NSCLC	RT+IT	SRS	IPI, NIVO or PEMBRO	19.6	1 year LC□84%
Pike et al., 2018^86^	Retrospective study	39	NSCLC	RT+IT	WBRT, SRS, WBRT+SRS	PEMBRO, NIVO or IPI	25.7	
Williams et al., 2017^100^	Phase I trial	16	Melanoma	RT+IT	WBRT *vs.* SRS	IPI	WBRT *vs.* SRS: 8, not reached	Concurrent ipilimumab 10 mg/kg with SRS is safe
Stokes et al., 2017^101^	Retrospective study	185	Melanoma	RT+IT	WBRT, SRS	Not specified	10.8	
Fang et al., 2017^102^	Retrospective study	137	Melanoma	RT+IT	SRS	Anti-CTLA-4 and/or anti-PD-1	16.9	
Petrelli et al., 2019^105^	Systematic review	1,520 (33 studies)	Melanoma (87%); NSCLC (11%); RCC (2%)	RT+IT	WBRT, SRS, WBRT+SRS	IPI (14 studies) PEMBRO (2 studies) anti-CTLA-4 and/or anti-PD-1 (16 studies)	15.9	1–2 year LC: 48% (523 patients), 31.6% (281 patients)
**RT+TT/IT**								
Choong et al., 2017^66^	Retrospective study	79	Melanoma	RT+TT/IT	SRS	Anti-CTLA4, anti-PD1 or BRAFi±MEKi	Anti-CTLA4, anti-PD1, BRAFi±MEKi: 7.5, 20.4, 17.8	Median iPFS: anti-CTLA4 (7.5 m), anti-PD1 (12.7 m), BRAFi±MEKi (12.7 m)
Gaudy-Marqueste et al., 2017^67^	Retrospective study	179	Melanoma	RT+TT/IT *vs.* RT alone	Gamma-Knife (GK)	Anti-CTLA/anti-PD2 and/or BRAFi±MEKi	1st GK *vs.* RT: 10.95, 2.29	

NR, not reported; BM, brain metastases; WBRT, whole brain radiotherapy; SRS, stereotactic radiosurgery; RT, radiotherapy; IT, immunotherapy; TT, target therapy; CHEMO, chemotherapy; OS, overall survival; iPFS, intracranial progression-free survival; LC, local control; ORR, objective response rate; m, months; IPI, ipilimumab; PEMBRO, pembrolizumab; NIVO, nivolumab; BRAFi, BRAF inhibitor; MEKi, MEK inhibitor.

## Conclusions and future perspectives

BMs are the most common intracranial tumors in adults, but conventional treatments show limited efficacy. RT has been demonstrated to control the intracranial progression of BMs. WBRT is a commonly recommended RT for multiple BMs that can decrease the risk of recurrence and new metastases; however, it can adversely affect neurocognitive function and QoL. Studies examining drugs and techniques to improve neurocognitive impairment *via* WBRT have yielded positive results. In contrast, as an alternative RT for surgery in patients with small, asymptomatic lesions and those with lesions that are not surgically accessible, SRS alone or after surgery can decrease cognitive impairment and toxic responses, while resulting in a prognosis similar to that with WBRT. Moreover, recent research has shown that the clinical efficacy of SRS is not inferior to that of WBRT for extensive BM. Finally, SRS is preferred as the first-line RT, if allowed after evaluation.

At present, most studies recommend RT postoperatively. However, the TTI between surgery and RT, and the dose fractionation of RT remain controversial. Hence, further studies should be conducted. Furthermore, ongoing clinical trials on neoadjuvant RT before surgery have shown promising results, thus providing a new perspective on the delivery of RT.

An increasing number of systemic treatments, particularly TT and IT, have demonstrated activity in the brain, and this modality might be reasonable for some patients, such as those with asymptomatic BMs, with initial systemic therapy instead of SRS or WBRT. Most treatment decisions are based on the primary tumor type.

Multidisciplinary treatment of BMs, including RT combined with systemic therapy, has shown promising results. Because of the low chemosensitivity of BMs and the poor penetration of chemical drugs into the BBB, chemotherapy alone is used not as a first-line treatment but as an addition to local treatment. Most chemical drugs are not effective against BMs, and only several of them, such as TMZ, improve patient survival when added to RT. Moreover, TT shows better effects.

RT and TT have been found to show mutually enhanced effects in pre-clinical studies; TT functions as a radiosensitizer, thus indicating that this combination shows promise in BM treatment. However, the existing retrospective and clinical trials have not reached a consensus. Research has focused primarily on BMs from NSCLC, whereas very few phase III clinical trials have been performed; the existing trials have yielded negative results and have focused on first-generation drugs. With the development of targeted drugs, more research is urgently needed to determine the efficacy of different drugs combined with different RT methods in patients with various mutations. For BMs from other cancers, such as BC and multiple myeloma, limited and low-level studies have suggested a promising outlook that urgently must be explored.

RT and IT have synergistic effects not only in the ITME but also at distant sites. At present, RT combined with IT is used primarily as a treatment for BMs from melanoma and lung cancer. Systematic reviews of the current literature have affirmed the safety and role of IT combined with RT, particularly SRS, in patients with BMs. Furthermore, retrospective studies have suggested that IT added to RT as treatment for BMs may be associated with a decreased incidence of new BMs and with favorable survival outcomes, without increased rates of adverse events. However, rigorous and reliable data from clinical trials are currently lacking and are urgently needed to support the efficacy of RT combined with systemic treatment modalities to treat BMs. Moreover, additional studies are needed to explore the mechanisms of central permeability and drug-radiation synergy. In addition, more immune-related approaches such as immune checkpoint blockers, CD47 blockers, cancer vaccines, and T cell therapy, should be investigated as new options.

In the future, stratified studies of combination therapy should be based on the number of BMs, the pathological type of the primary tumor, and the genotype of the primary tumor, as well as the metastatic foci. Furthermore, treatment administration, such as the best method for administering RT and systemic drugs, the optimal dose, and the drug order, are needed to achieve individualized, multidisciplinary, and precise treatment.
